# Vitamin D and bone health

**DOI:** 10.1186/1471-2474-16-S1-S16

**Published:** 2015-12-01

**Authors:** Susan Lanham-New

**Affiliations:** 1 Department of Nutritional Sciences, Faculty of Health and Medical Sciences, University of Surrey, Guildford, GU2 7XH, UK

## 

Throughout the life-cycle, the skeleton requires optimum development and maintenance of its integrity to prevent fracture. Bones break because the loads placed upon them exceed the ability of the bone to absorb the energy involved. It is now estimated that 1:3 women and 1:12 men over the age of 55 years will suffer from osteoporosis in their lifetime and in the UK, at a cost in excess of £1.7 billion per annum to the exchequer. The pathogenesis of osteoporosis is multi-factorial. Both the development of peak bone mass and the rate of bone loss are determined by key endogenous and exogenous factors. Calcium supplements appear to be effective in reducing bone loss in late menopausal women (>5 years post-menopause), particularly in those with low habitual calcium intake (< 400mg/d). In younger postmenopausal women, who are not vitamin D deficient, vitamin D supplementation has little effect on BMD. However, vitamin D and calcium supplementation studies have been shown to reduce fracture rates in the institutionalized elderly but there remains controversy as to whether supplementation is effective in reducing fracture in free-living populations. Re-defining vitamin D requirements in the UK is urgently needed since there is evidence of extensive hypovitaminosis D in the UK. Low vitamin D status is associated with an increased risk of falling and a variety of other health outcomes and is an area that requires urgent attention. The role of other micronutrients on bone remains to be fully defined, although there are promising data in the literature for a clear link between vitamin K nutrition, dietary protein and dietary alkali on skeletal integrity including fracture reduction.

